# Tiny but mighty: Diverse functions of uORFs that regulate gene expression

**DOI:** 10.1016/j.csbj.2024.10.042

**Published:** 2024-10-28

**Authors:** Zhenfei Zhong, Yajie Li, Qinmiao Sun, Dahua Chen

**Affiliations:** a Institute of Biomedical Research, Yunnan University, Kunming, Yunnan 650500, China; b State Key Laboratory of Membrane Biology, Institute of Zoology, Chinese Academy of Sciences, Beijing 100101, China; c Key Laboratory of Organ Regeneration and Reconstruction, Beijing 100101, China; d Beijing Institute for Stem Cell and Regenerative Medicine, Beijing 100101, China; e School of Life Sciences, University of Chinese Academy of Sciences, Beijing 100049, China; f Southwest United Graduate School, Kunming 650500, China

**Keywords:** UORFs, Microbes, Translation, Transcription, Gene expression and regulation, Stress response

## Abstract

Upstream open reading frames (uORFs) are critical *cis*-acting regulators of downstream gene expression. Specifically, uORFs regulate translation by disrupting translation initiation or mediating mRNA decay. We herein summarize the effects of several uORFs that regulate gene expression in microbes to illustrate the detailed mechanisms mediating uORF functions. Microbes are ideal for uORF studies because of their prompt responses to stimuli. Recent studies revealed uORFs are ubiquitous in higher eukaryotes. Moreover, they influence various physiological processes in mammalian cells by regulating gene expression, mostly at the translational level. Research conducted using rapidly evolving methods for ribosome profiling combined with protein analyses and computational annotations showed that uORFs in mammalian cells control gene expression similar to microbial uORFs, but they also have unique tumorigenesis-related roles because of their protein-encoding capacities. We briefly introduce cutting-edge research findings regarding uORFs in mammalian cells.

## Graphical Abstract

**Figure:**
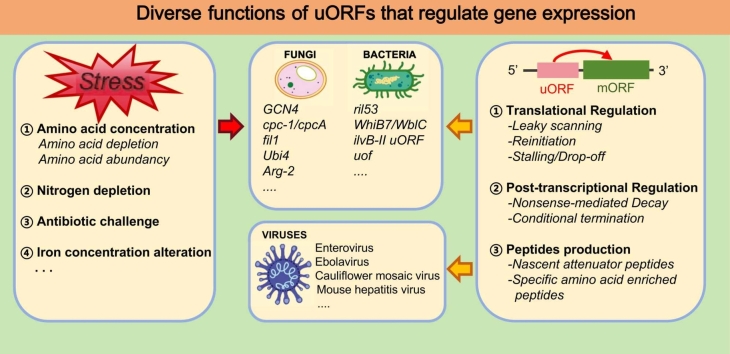

## 1 Introduction

**Fig. 1:**
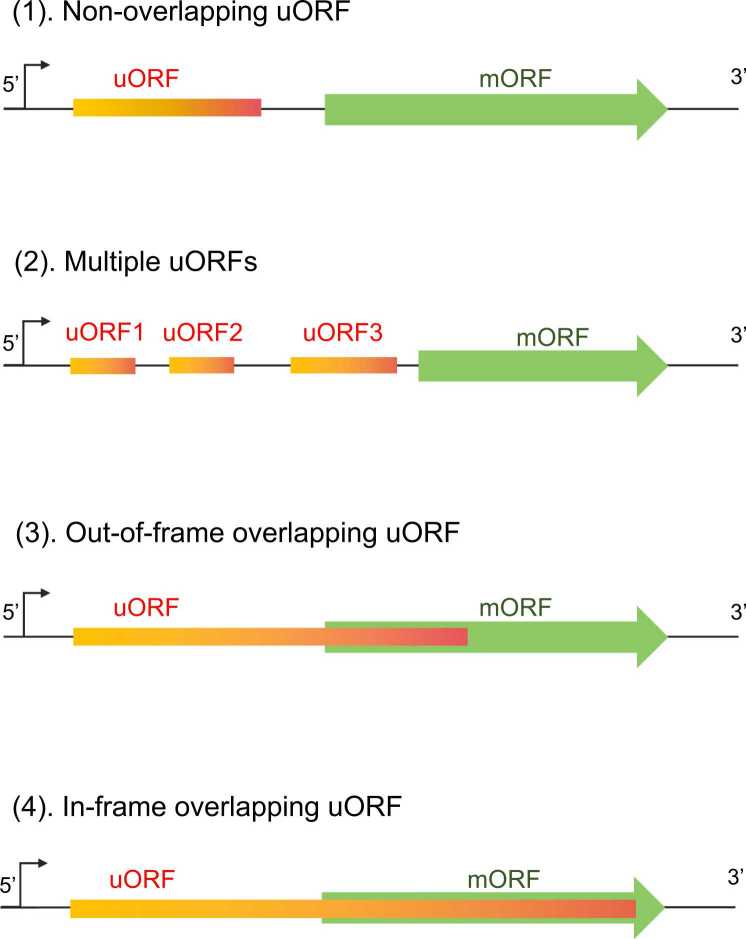
Illustration of different uORF types. (1) Non-overlapping uORFs, (2) multiple uORFs, (3) out-of-frame overlapping uORFs, and (4) in-frame overlapping uORFs.

Gene expression regulation is critical for the adaptability of living organisms in response to environmental changes and internal stress [1], [2], [3], [4], [5], [6]. Gene expression is regulated at various levels (e.g., epigenetic, transcriptional, translational, and other levels), which involves the effects of different signaling cascades and a series of mediators that synergistically respond to stimuli [7], [8], [9], [10], [11], [12]. Gene expression regulated by an upstream open reading frame (uORF), which occurs at the translational level or post-transcriptional level, represents a relatively straight-forward and energy-efficient process. A uORF is a short sequence containing a start codon, stop codon, and at least one sense codon in frame and is located upstream of the primary gene coding region. Although uORFs were first identified in microbes, wherein they contribute to a mechanism that suppresses translation [13], they were subsequently revealed to be common in microbes as well as higher eukaryotes, including *Arabidopsis thaliana* [14], maize [15], *Drosophila melanogaster* [16], [17], zebrafish [18], [19], [20], mouse [21], and human [22], [23]. Approximately 20 % of fungal mRNAs contain uORFs, but the percentage of plant and mammalian mRNAs with uORFs is even higher [24], as indicated by the accumulating translatomic data generated from genome-wide ribosome profiling analyses. The common structural features of these uORFs have been confirmed. Compared with canonical protein-coding ORFs and main or primary ORFs (mORFs or pORFs), uORFs are considerably shorter, with a median size of 22 codons [25]. Moreover, the translation efficiency of uORFs is lower than that of mORFs [26]. Because the start codons of uORFs are in the 5'-untranslated region (UTR) of the mORF, they are classified into different types on the basis of the position of their stop codons relative to mORFs: non-overlapping uORFs whose stop codons are upstream of mORF start codons and a non-overlapping uORF subtype with multiple uORFs, which exist in approximately 20 % of mammalian mRNAs, each with separate or synergistic functions [27]; out-of-frame and in-frame overlapping uORFs (Fig. 1). Unlike out-of-frame uORFs that disrupt the production of mORF-encoded proteins, in-frame uORFs result in mORF-encoded proteins with N-terminal extensions, similar to protein isoforms. In this review, we mainly focus on non-overlapping uORFs to illustrate their regulatory effects on gene expression.

## 2 Regulatory effects of uORFs on mORF expression

According to previous studies, uORFs function at the translational level by interfering with mORF translation initiation [28], [29] or at the post-transcriptional level by mediating mRNA degradation via nonsense-mediated mRNA decay (NMD) [30]. In addition, some uORFs encode small bioactive peptides (micropeptides or upstream conserved coding regions) to regulate mORF translation [28].

**Fig. 2:**
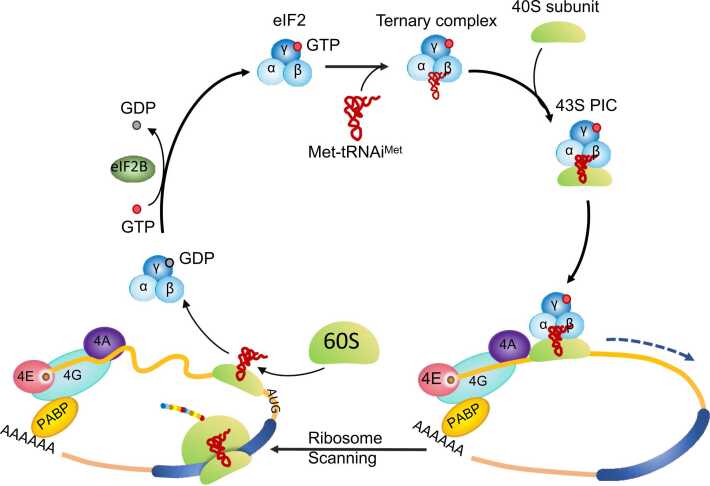
Translation initiation in eukaryotes. A complete translation process includes translation initiation, elongation, termination, and ribosome recycling. During translation initiation, the 40S subunit binds to the ternary complex (TC) consisting of eIF2, GTP, and methionine initiator transfer RNA (Met-tRNA_i_^Met^) along with eIF5 to form the 43S PIC. The binding of PIC to mRNA is facilitated by the eIF4 complex. The eIF4 complex, which consists of the cap-binding protein eIF4E, the scaffolding protein eIF4G, and the RNA helicase eIF4A, recruits ribosomes to mRNA. The mRNA-binding PIC then slides downstream of the mRNA, scanning for a start codon. After the translation start site is selected, eIF2 promotes GTP hydrolysis and GDP dissociates from mRNA. The 60S subunit is then recruited to PIC to form the 80S initiation complex (IC) for elongation. During elongation, the ribosome moves along the mRNA to synthesize the nascent peptide according to each codon. Translation termination occurs when the ribosome encounters a stop codon, after which the newly synthesized polypeptide is released and the ribosome dissociates and is recycled.

Translation initiation represents a critical point for translation regulation [31]. uORFs impede mORF translation initiation through various mechanisms. For example, they disrupt the interaction between ribosomes and start codons/stop codons and contribute to leaky scanning and translation reinitiation (REI). Translation is initiated when a ribosome recognizes the start codon via ribosome scanning (Fig. 2) [32], [33], [34], [35], [36]. The uORF start codon can serve as a translation start site (TSS) and interfere with translation initiation. In the presence of multiple AUG codons or near-cognate codons, such as CUG or UUG, the TSS is determined by both the sequence context flanking the start codons and the relative concentration of the initiation factors eIF1 and eIF5. Start codons within Kozak sequences are optimal for initiating translation [37], [38]. Moreover, high eIF1 levels favor the initiation of translation at start codons with a strong sequence context, while high eIF5 levels tend to result in the initiation of translation at start codons with a weak sequence context [37], [38], [39]. The uORF start codon and the factors mentioned above cooperatively regulate gene expression. An example of this cooperation is the regulated expression of *eIF5*, which harbors uORFs with start codons in a poor context. Under stringent start codon selection conditions, *eIF5* uORFs are suppressed, resulting in enhanced *eIF5* expression. This, in turn, favors the weak start codon of the *eIF1* mORF, which increases eIF1 production and enhances the positive feedback loop of *eIF5* translation [40], [41], [42]. Previous research showed that uORF start codons are usually surrounded by less favorable contexts than mORF start codons [20], [43]. Under stringent start codon selection conditions, uORF start codons are likely to be bypassed by the scanning ribosome, but there are a few exceptions [41]. These observations suggest that most uORFs regulate gene expression by serving as *cis*-acting elements rather than through their encoded peptide. This has been confirmed by studies that demonstrated that mORF expression is enhanced when uORFs are blocked [44], [45], [46], [47], [48].

**Fig. 3:**
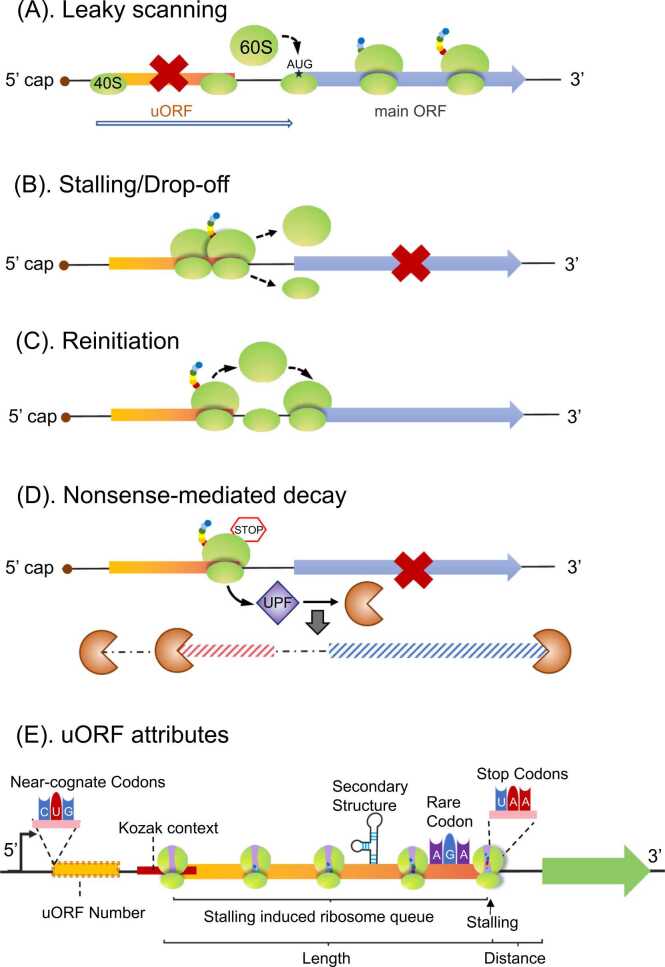
Regulatory functions of uORFs. (A) Leaky scanning. When the uORF start codon is not selected as a translation start site, PIC is expelled from the uORF stop codon and reassembled at the mORF start codon to initiate translation. (B) Ribosome stalling/drop-off. The uORF stop codon mediates uORF translation termination, which leads to the separation of the 40S and 60S subunits from the mRNA and repressed mORF translation. (C) Translation reinitiation. The 40S subunit attaches to the mRNA following uORF translation termination. The 60S subunit is then recruited to the mORF start codon to restart mORF translation. (D) Nonsense-mediated mRNA decay. uORF translation termination at the stop codon triggers nonsense-mediated mRNA decay facilitated by UPF proteins, which is followed by the recruitment of an exonuclease or endonuclease that degrades mRNA. (E) Factors contributing to uORF inhibitory efficiency. The start codon, stop codon, codon usage, uORF length, number of uORFs, Kozak sequence context around the start codon, uORF-encoded peptides, distance from the mORF, and uORF secondary structures contribute to uORF effects on mORFs.

During translation termination, the 60S subunit disassembles from the translation complex for a new round of translation when the stop codon is reached in a procedure known as ribosome recycling [49], [50], [51], [52]. Stop codons of uORFs regulate mORF translation by mediating ribosome drop-off or REI. Different stop codons vary in terms of termination efficiency, with UAA being the most efficient, followed by UAG and UGA. Notably, uORFs with a strong stop codon can effectively inhibit mORF translation [53]. If uORF start codons are not selected as a TSS, the 40S subunit will slide past the uORF (no translation) in a process known as leaky scanning, thereby enabling the undisturbed translation of the downstream mORF (Fig. 3A). The selection of a uORF start codon as a TSS and the presence of a strong uORF stop codon will lead to ribosome stalling or drop-off (Fig. 3B), resulting in inhibited mORF translation. In addition, uORFs compete with mORFs for the eIFs required for the assembly of a pre-initiation complex (PIC), resulting in downregulated mORF expression.

A translation termination signal at the stop codon of uORFs leads to ribosome disassembly and a pause in translation, necessitating REI for mORF expression (Fig. 3C). Although the mechanism underlying REI has not been comprehensively characterized, the 40S subunit must attach to mRNA together with eIF3 and eIF4 for mORF REI [54]. REI is affected by multiple factors, including the distance between the uORF and mORF as well as uORF length. The length of uORFs also influences REI efficiency because eIFs tend to be lost during the translation of relatively long uORFs, but a uORF that is too short adversely affects 60S subunit recruitment [55]. Accordingly, uORF-mediated REI is an inherently variable and highly context-dependent process, especially in mORFs that are regulated by multiple uORFs.

Some uORFs regulate mORF expression via their encoded micropeptides. A well-studied model revealed that a uORF-encoded nascent peptide induces ribosome stalling, which is followed by suppressed mORF translation, or mRNA degradation via NMD in response to various stimuli, including changes to nutrient, metabolite, and antibiotic levels [56]. Because of limited space, we will only briefly introduce (1) uORF-encoded arginine attenuator peptide (AAP)-regulated arginine synthesis in response to arginine availability and (2) uORF-mediated polyamine synthesis and transport in response to different polyamine concentrations as representative examples of the regulatory effects of uORF-encoded peptides on gene expression in this review.

Some uORFs regulate mORF expression by inducing NMD (Fig. 3D), which is a translation-dependent mechanism triggered by a premature termination codon (PTC); the stop codon of uORFs can serve as a PTC and mediate mRNA degradation [57]. Furthermore, uORFs have a general suppressive effect on mORF translation through codon usage. Specifically, compared with mORF codons, uORF codons are poorly conserved and uORFs with rare triplet codons increase mORF repression because of the associated decrease in the ribosome scanning rate or induced stalling [26], [58]. In addition, a mutation that converts rare codons to common codons encoding the same amino acids in uORF2 of a methionine synthase gene completely abolishes the ability of the uORF to inhibit translation [59]. These observations suggest that codon usage contributes to uORF-mediated mORF translation inhibition.

## 3 Factors contributing to the efficiency of uORF inhibitory effects

The regulatory effects of different uORFs on mORF expression vary among genes. Similarly, how efficiently they regulate gene expression also varies. Increasing studies on mutated uORFs revealed the factors contributing to uORF functions.

The functions of uORFs are primarily determined by their start and stop codons as well as their position relative to that of mORFs. The uORF start and stop codons regulate mORF expression by competing for TSS and mediating ribosome drop-off or REI as described above. uORFs with strong start or stop codons have relatively high suppressive effects on mORF expression. Mutating the uORF start codon reportedly increases the expression of the *eIF5* mORF [40]. Similarly, uORFs with a strong stop codon also have high repressive effects on mORF expression via the inhibition of REI [53]. Other features of uORFs (e.g., length, number, distance from mORFs, and uORF-related secondary structure) also contribute to the efficiency of uORF effects (Fig. 3E). Increases in uORF length enhance the repression of mORF expression, as evidenced by the observed increase in uORF-mediated inhibition of mORF expression when the uORF was extended by the insertion of HaloTag-GFP [60]. The human immunodeficiency virus *tat* sequence is located upstream of *rev* and *nef* in the same mRNA. Inserting a premature stop codon in *tat* leads to increased *rev* and *nef* expression, whereas increasing the length of *tat* results in decreased *rev* and *nef* expression [61]. This inverse correlation between the length and uORFs suppression is also confirmed in zebrafish, mouse and human by ribosome profiling [20]. Moreover, uORF length is negatively correlated with mORF REI efficiency [62]. The expression of the hepatitis B virus polymerase gene *P* is regulated by its uORF *Cd2*; an increase in *Cd2* length reportedly decreases *P* REI [63]. In yeast, long uORFs increase NMD more than short uORFs [64]. The efficiency of uORF functions is also affected by the distance between the uORF stop codon and the mORF start codon. An increase in the distance between the uORF and mORF is positively correlated with mORF REI efficiency [65]. Additionally, uORFs distal to mORFs increase NMD more than uORFs adjacent to mORFs [64].

The functions of some uORFs are auto-regulated in response to the availability of essential nutrients (e.g., polyamine). Ornithine decarboxylase (ODC), which catalyzes the first step of the polyamine synthesis pathway, is regulated by an antizyme (OAZ1) that binds to ODC and promotes ODC degradation [66]. OAZ1 production is induced by extensive polyamine-regulated ribosomal frameshifting. The OAZ1 function is negatively regulated by AZIN1 [66]. *AZIN1* contains a suppressive uORF, and this uORF encodes the peptide that harbors a highly conserved C terminus with a PPW sequence. Under low polyamine conditions, leaky scanning suppresses uORF translation and increases *AZIN1* translation, thereby enhancing polyamine synthesis. Sufficient polyamine levels induce ribosome stalling at the PPW site and suppresses *AZIN1* translation. Specifically, polyamine inhibits eIF5-facilitated PPW synthesis during the occasional translation of uORF, which induces ribosome stalling at the PPW site. Notably, this stalling causes the following scanning ribosomes to queue at the pausing site. This eventually positions a ribosome at the uORF start codon, leading to enhanced uORF translation, which subsequently enhances ribosome stalling [39]. Hence, polyamine interacts with the uORF to form a positive feedback loop that restricts *AZIN1* expression in response to polyamine repletion [39]. Interestingly, a similar regulatory mechanism controls the expression of the gene encoding the polyamine importer Hol1. *HOL1* contains a uORF in its 5′-UTR that harbors a conserved PS* motif (* represents a stop codon). Leaky scanning of the uORF results in increased *HOL1* translation under low polyamine conditions, but high polyamine levels interfere with eIF5 and impairs the release of the *HOL1* uORF-encoded peptide in the PS* motif, leading to a ribosome queue and further repression of *HOL1* translation [67]. In addition, similar patterns of conserved sites in uORF-encoded peptides controlling mORF translation were also identified during analyses of uORF-regulated expression of *CHOP*, *GADD34*, and *CReP* in an integrated stress response (ISR) [68], [69].

The efficiency of uORF functions is affected by the number of uORFs in the same transcript. In *Saccharomyces cerevisiae*, *GCN4* contains four uORFs, of which the first two mediate *GCN4* REI, while the third and fourth are REI-non-permissive uORFs, cooperating in a fail-safe manner to respond to stress [70]. Similarly, the expression of *ATF4*, which is the mammalian homolog of *GCN4*, is also regulated by its two uORFs in a process influenced by the eIF2α phosphorylation level in an ISR [71].

Certain uORFs are involved in the formation of secondary structures (e.g., stem-loop) that decrease the uORF translation rate as well as mORF REI. The mRNA secondary structure is involved in multiple gene expression regulatory mechanisms (e.g., attenuator and anti-terminator formation as well as ribosome shunting); this will be discussed in more detail in the next section. In addition, elongation delay due to the secondary structure decreases the probability that the factors necessary for REI are retained when the 40S subunit resumes scanning, thereby adversely affecting REI efficiency [44]. In accordance with this finding, modulating the uORF secondary structure can decrease mORF REI [72].

## 4 Molecular mechanisms underlying the regulatory effects of uORFs on mORFs in microbes

Although uORF-mediated regulation of gene expression represents a critical and universal mechanism induced by cell stress and various environmental stimuli, the precise effects of uORFs on mORFs and the underlying molecular mechanisms vary greatly in different genes and contexts. In the subsequent sections, we provide an overview of several thoroughly investigated microbial uORFs and the associated mechanisms, thereby comprehensively elucidating uORF functions related to gene expression regulation.

### 4.1 uORFs play a crucial role in fungal stress responses

**Fig. 4:**
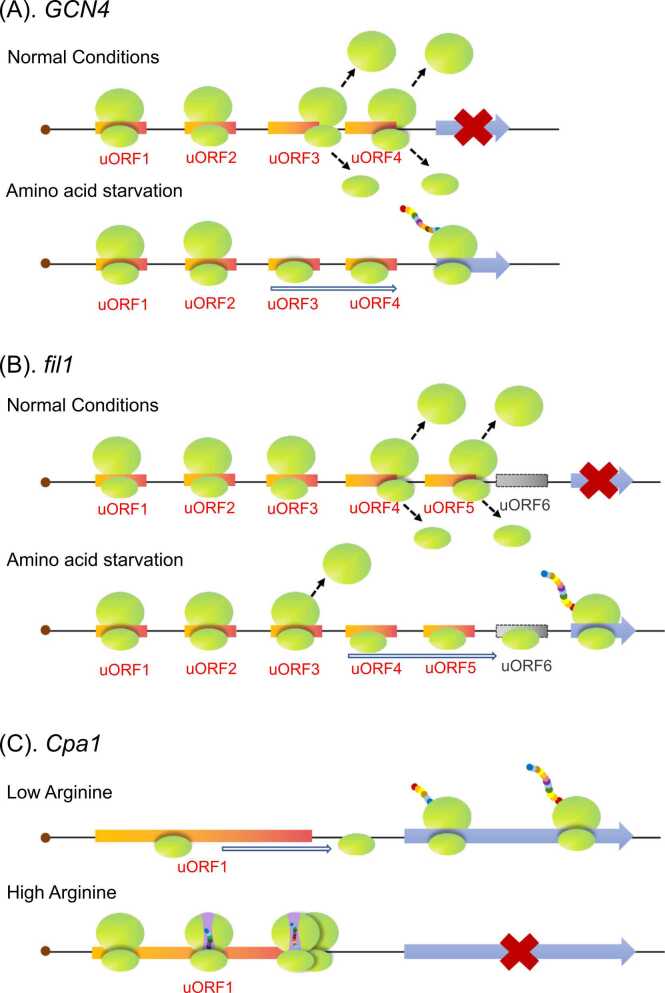
uORF functions in fungal stress responses. (A) uORFs regulate *GCN4* expression. Under normal conditions, uORF1 translation leads to uORF4 translation, which then decreases *GCN4* translation because of relatively poor REI. Under amino acid starvation conditions, TC insufficiency hampers 40S ribosomal subunit recruitment and translation reinitiation at uORF4, causing the ribosome to bypass uORF4 and reinitiate translation at the *GCN4* start codon. (B) uORFs regulate *fil1* expression. Under normal conditions, *fil1* expression is repressed by the translation of uORF4 and 5. In response to amino acid starvation, decreased TC levels cause the 40S ribosomal subunit to bypass repressive uORF4 and 5, thereby mediating *fil1* translation. (C) uORFs regulate *Cpa1* expression. When the arginine concentration is low, leaky scanning suppresses uORF translation and promotes *Cpa1* expression. Conversely, high arginine levels induce nascent peptide conformational changes that lead to ribosome stalling and inhibit *Cpa1* translation.

In *S. cerevisiae*, the expression of *GCN4*, which encodes a critical transcription factor for stress responses, is regulated by eIF2 and its four uORFs. Functional analyses indicated uORF1 and 2 mediate *GCN4* REI, while uORF3 and 4 repress *GCN4* REI [73]. Because the coordinated effects of uORF1 and 4 are sufficient for regulating *GCN4* expression, both uORFs were included in an analysis of the uORF-mediated regulation of *GCN4* expression [73]. Under normal conditions, uORF1 translation transitions to uORF4 translation with sufficient production of the ternary complex (TC), after which uORF4 suppresses *GCN4* REI. Under amino acid starvation conditions, the stress-responsive kinase Gcn2 is activated and phosphorylates eIF2α, which converts eIF2 from a substrate to an inhibitor of eIF2B. This leads to decreased TC assembly and hampers 40S ribosome recruitment for uORF4 translation [73]. Consequently, ribosomes bypass the uORF4 start codon and recognize the *GCN4* start codon (Fig. 4A). *cpc-1* and *cpcA* are homologs of *S. cerevisiae GCN4* in *Neurospora crassa* and *Aspergillus nidulans*, respectively. Consistent with *GCN4* expression, the expression of *cpc-1* and *cpcA* is also regulated by uORFs in their 5′-UTRs in response to the depletion of amino acids [74].

Similar to *GCN4* and *ATF4*, *fil1* encodes a stress-responsive transcription factor and its expression is regulated by six uORFs. The transition from uORF1 translation to uORF3 translation mediates *fil1* REI, whereas the translation of uORF4 and 5 fully represses *fil1* expression. An exposure to histidine starvation conditions decreases TC formation and causes the 40S ribosome to bypass repressive uORF4 and 5, thereby enhancing *fil1* translation (Fig. 4B) [75]. In *Schizosaccharomyces pombe*, the expression of *Ubi4*, which encodes a mediator of responses to high temperatures, starvation, and oxidative stress [76], is regulated by its uORFs under stress conditions. *Ubi4* translation is activated when nitrogen is depleted, which coincides with a decrease in the translation levels of its two uORFs, suggestive of a potential regulatory effect of uORFs on *Ubi4* translation under nitrogen-depleted conditions [76].

In response to environmental stimuli, some uORFs regulate mORF expression through their encoded peptides. *N. crassa Arg-2* and its ortholog in *S. cerevisiae* (*Cpa1*) encode the small subunit of carbamoyl-phosphate synthetase, which facilitates arginine synthesis [77]. *Cpa1* expression is regulated by uORFs encoding an AAP in response to arginine concentration changes. When arginine is depleted, *Cpa1* expression ensures arginine production, while the expression of its upstream AAP-encoding uORF is repressed because of leaky scanning (Fig. 4C) [78]. Conversely, increases in arginine levels induce a conformational change in the nascent AAP within the ribosome exit tunnel, resulting in ribosome stalling at the uORF stop codon and inhibited *Cpa1* translation [79]. Moreover, AAP-induced ribosome stalling also triggers NMD of *Cpa1* mRNA [30]. Furthermore, AAP functions depend on conserved amino acid sequences, but the position of the conserved amino acids in the peptide does not affect function [80]. Further research showed that the amino acid sequences responsible for arginine-induced conformational changes are highly conserved in AAPs in many fungi [81]. In addition to regulating gene expression in response to nutrient supply, uORFs also regulate gene expression during unfolded protein responses (UPRs), as shown by the regulated expression of the gene encoding HIT nucleotide-binding 1 (*HNT1*) [82].

### 4.2 uORFs regulate gene expression in bacteria

The coupling of transcription and translation is a hallmark of bacterial mRNAs, enabling the co-transcriptional regulation of translation attenuation or transcription termination [83], [84], [85]. In response to metabolic stress and antibiotics, uORFs modulate the formation of attenuator or anti-terminator mRNA secondary structures, thereby regulating gene expression.

The regulatory effects of uORFs on bacterial antibiotic resistance (i.e., by mediating conditional termination) were revealed by Term-seq, a quantitative sequencing method for determining the transcription termination site of bacterial RNA [86]. Term-seq data indicated that lincomycin resistance is mediated by *lmo0919*, whose expression is regulated by the uORF *rli53* in *Listeria monocytogenes*. *rli53* forms an attenuator in the absence of lincomycin, leading to the premature termination of the transcription of the downstream gene *lmo0919*. Following the addition of lincomycin, *rli53* adopts an anti-terminator structure, enabling the expression of *lmo0919*, which encodes an antibiotic efflux pump [86]. In addition, uORFs contribute to conditional premature termination, thereby regulating *Mycobacterium tuberculosis* pathogenicity [87].

**Fig. 5:**
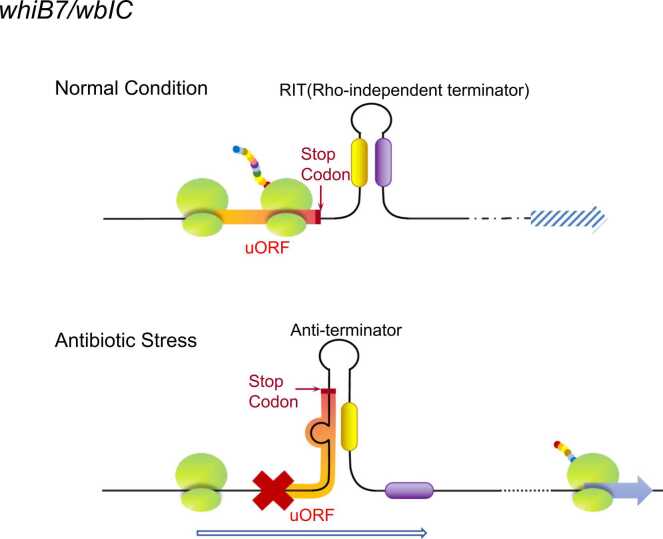
Regulatory effects of uORFs in bacteria. In the absence of antibiotics, the *whiB7/wblC* uORF is completely translated, which suppresses *whiB7/wblC* transcription and promotes Rho-independent terminator (RIT) formation, resulting in premature transcriptional termination. Antibiotic stress conditions inhibit uORF translation by favoring anti-terminator formation, which is conducive to *whiB7/wblC* transcription.

WhiB7 and WblC (WhiB-like protein C in *Streptomyces* species) are conserved transcription factors in actinomycetes that confer resistance to multiple antibiotics [88], [89], [90], [91], [92]. Earlier studies showed uORFs mediate *whiB7/wblC* transcriptional attenuation in response to antibiotics [93], [94], [95], [96], [97], [98]. In the absence of antibiotics, *wblC* transcription is suppressed by uORF translation, which promotes the formation of a Rho-independent terminator (RIT), leading to premature transcriptional termination. In the presence of antibiotics, uORF translation is disrupted, resulting in the formation of an anti-terminator, which facilitates the read-through for *wblC* transcription (Fig. 5). Moreover, uORF-regulated *whiB7* and *wblC* expression also occurs under amino acid starvation conditions [98], [99].

Acetohydroxyacid synthase I (AHAS) and isomeroreductase (IR) have critical functions for the biosynthesis of three branched-chain amino acids (BCAAs), namely isoleucine (I), leucine (L), and valine (V). Both AHAS and IR are encoded by the *ilvBNC* operon in *Corynebacterium* species. A structural analysis of *ilvB* in *Corynebacterium ulcerans* revealed that when the amino acid supply is sufficient, the *ilvB-II* motif, which contains a conserved short ORF, suppresses mORF translation through terminator stem formation. The terminator is converted to an anti-terminator stem that enables downstream translation in response to the availability of aminoacylated BCAA tRNAs. The *ilvB-II* uORF regulates *ilvB* expression because it encodes a functional peptide enriched with BCAAs. When BCAAs are abundant, the *ilvB-II* uORF is translated quickly, which prevents the formation of an anti-terminator structure. In contrast, insufficient amounts of BCAAs decrease the uORF translation rate, which is conducive to terminator stem formation, ultimately leading to transcriptional termination [100], [101], [102]. This transcription attenuation mechanism involving the *ilvBNC* operon is consistent with the regulated expression of tryptophan biosynthesis genes, indicating that uORF-mediated terminator or anti-terminator formation is a conserved gene expression regulatory mechanism responsive to environmental changes [103].

Another example of uORF-regulated post-transcriptional gene expression is the expression of the *Escherichia coli* gene *fur* (ferric uptake regulator), which plays a key role in iron metabolism [104]. The *fur* uORF, which is designated as *uof*, keeps *fur* expression at a relatively constant level to maintain iron homeostasis. The *uof-fur* transcript level is regulated by the non-coding RNA RyhB targeting *uof* in response to the iron concentration. The expression of *fur* leads to the formation of metallo-Fur, which binds to DNA to inhibit *ryhB* and *uof-fur* transcription. Iron depletion activates RyhB production, which results in the degradation of *uof-fur* mRNA and the mRNA encoding other iron-binding proteins [105]. Iron depletion also decreases the decoding efficiency or even ribosome stalling on *uof* transcripts that harbor multiple rare codons [105]. The coupled expression of mORFs and uORFs containing iron-responsive rare codons has also been detected in *Shigella* species, *Pseudomonas aeruginosa*, and *Vibrio cholerae*, implying there may be a universal uORF-based mechanism regulating iron metabolism in bacteria [105].

### 4.3 Regulatory effects of uORFs in viruses

As obligate intracellular parasites, viruses have relatively compact genomes, but uORFs are present in viral genomes, suggesting they have important roles related to viral survival, replication, or pathogenicity. The virus life cycle depends largely on host cells, and viral uORF functions are regulated by mechanisms similar to those in host cells.

Enteroviruses are ubiquitous pathogens in mammals. According to earlier research, UP, which is a functional protein encoded by a uORF conserved in major enterovirus groups, helps these viruses infect gut epithelia [106]. An analysis of echovirus 7 (EV7) suggested that the uORF-encoded polypeptide UP is unnecessary for viral replication, but it disrupts membranes to promote viral release and is a key facilitator of viral growth in gut epithelial cells [106]. Moreover, changes to the UP amino acid sequence have been detected in enterovirus A71 (EVA71) and coxsackievirus A2, which were associated with the 2018 enterovirus outbreak, indicating uORFs may influence EVA17 pathogenicity [107].

Ebolaviruses (EBOVs) are RNA viruses that harbor long non-coding regions at their 5′ and 3′ ends. The 5′-UTR of EBOV genes *VP35*, *VP30*, *VP24*, and *L* contain uORFs. Among these genes, *L* encodes the catalytic subunit of the EBOV polymerase required for viral RNA synthesis, but the excessive accumulation of L has detrimental effects on polymerase activity. *L-uORF* (ORF upstream of *L*) suppresses *L* expression by serving as a TSS. Under cell stress conditions, eIF2 phosphorylation interferes with *L-uORF* translation and upregulates *L* expression. A mutated *L-uORF* leads to attenuated EBOV replication, reflecting the importance of uORFs for viral replication [108].

**Fig. 6:**
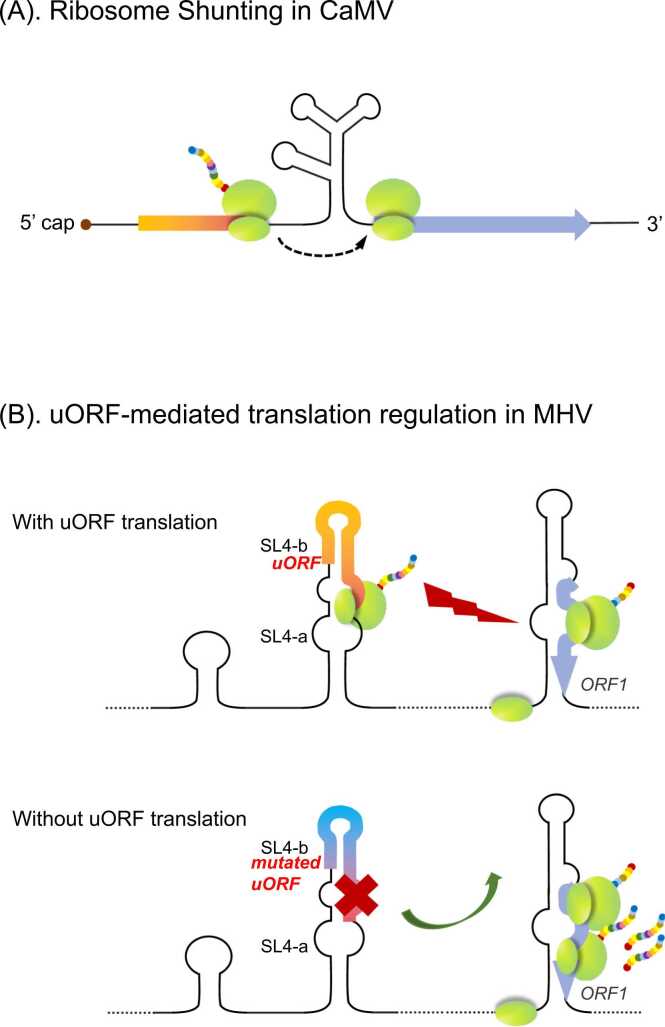
Regulatory effects of uORFs in viruses. (A) Ribosome shunting in CaMV. After the first uORF is translated, the ribosome moves directly to the region downstream of the looped structure on the 35S leader, bypassing the regions in the stem structure. (B) uORF-mediated translation regulation in MHV. Under normal conditions, the uORF in the SL4b stem-loop is translated and ORF1 translation is attenuated. A mutation in the uORF within SL4b inhibits uORF translation, but increases ORF1 translation.

Ribosome shunting is an unconventional uORF-associated regulatory mechanism that serves as an alternative to linear scanning on a hairpin configuration. Ribosome shunting has been extensively studied in the cauliflower mosaic virus (CaMV) [109], [110]. There are six uORFs located in the 5′-UTR of the pre-35S RNA of CaMV, with a hairpin loop structure downstream of the first uORF [110], [111]. The ribosome moves directly to the region downstream of the looped structure after the translation of the first uORF, bypassing the regions in the stem structure (Fig. 6A); this process has been designated as ribosome shunting. Similar shunting mechanisms were detected in other viruses from the Caulimoviridae genera such as rice tungro bacilliform virus, RNA picorna-like virus such as rice tungro spherical virus, and adenovirus [112], [113], [114]. Moreover, analysis in adenovirus infection showed that ribosome shunting retains viral gene expression in translation inhibition mediated by eIF4E during the late stage of infection or cell heat shock [113], [114].

The 5′-UTR of the coronavirus mouse hepatitis virus (MHV) contains four stem-loop (SL) structures that regulate the expression of the downstream ORF1. The second base-paired region of SL4 (SL4b) contains a small uORF that is present in most coronaviruses, wherein it is associated with the phylogenetically conserved SL4. A reverse genetics analysis revealed that SL4 serves as a spacer facilitating viral replication [115]. A mutation to the uORF that inhibits ORF1 REI leads to increased ORF1 translation (Fig. 6B). This uORF regulates ORF1 expression, thereby maintaining the optimal amount of the nonstructural protein product for viral viability [115], [116], [117]. These results suggest that uORFs are potentially beneficial for the adaptation of viruses, including invasion of host cells, replication during cell stress.

## 5 uORFs regulate gene expression in mammalian cells

Compared with the corresponding mechanisms in various microbes, more sophisticated mechanisms are required for the regulation of gene expression at the translational level in higher eukaryotes [7], [8], [9], [10], [11], [12]. However, increasing evidence of uORF-regulated gene expression in mammalian cells in response to cellular stress [118], [119] suggests uORFs are essential for the regulation of mammalian gene expression. Mammalian cells are exposed to various stresses, including oxidative stress, hypoxia, unfolded protein-induced ER stress, and metabolic stress, and failing to promptly respond to these stress conditions leads to cell death, tissue dysregulation, and tumorigenesis. How cells deal with these stresses has been studied extensively, but the role of uORFs in cellular stress responses and their effects on human diseases remain to be thoroughly investigated.

Translatomic data obtained from ribosome profiling analyses conducted using uORF annotation tools confirmed the widespread existence of uORF translation in mammalian cells, and also revealed that mammalian uORFs function similarly to their microbial counterparts, as shown in studies comparing the translational features of uORFs and mORFs in various mammalian samples based on translatomic data [21], [22], [23]. Moreover, annotation of uORF with expanded reference databases generated from mass spectra greatly contribute to uORF identification [120]. Meanwhile, functional assays of uORFs revealed detailed uORF-related mechanisms mediating mammalian cell stress responses [39], [68], [69], [121], [122], [123], [124]. New tools for RNA editing [125] and antisense oligonucleotide (ASO) targeting [126] have also enhanced the functional characterization of uORFs. Remarkably, the removal of the uORF upstream of *IRF6* during the Van der Woude syndrome-associated mutation via deaminase-enabled recoding of RNA (DECOR)-mediated RNA editing reportedly rescues *IRF6* expression [125]. Targeting uORFs with ASOs effectively upregulates mORF expression. Furthermore, a *GATA4* uORF-targeting ASO upregulates cardiac *GATA4* expression, which protects against cardiac hypertrophy [126], [127], [128]. These studies indicate targeting uORFs may be beneficial for treating diseases caused by changes in gene expression levels. Additionally, analyses of uORF mutations have also provided insights into cancer development. Jürgens and colleagues compared cancer-associated somatic mutations of uORFs, including uAUG, uStops (uORF stop codons), and aTISs (alternative translation initiation sites), and determined uORF variations were most frequent in *CHCHD2*, *FRG2C*, *HLA-DRB1*, and other genes [129]. Functional assays also confirmed that 19 of the identified variants regulated mORF translation [129]. Other studies showed that the expression levels of cancer-related genes, such as *PD-L1*, *HER2*, and *BRCA1*, are regulated by uORFs [130], [131], [132]. These uORFs also encode proteins that affect tumorigenesis. The *PTEN* uORF-encoded protein MP31 inhibits tumorigenesis and sensitizes glioblastoma (GBM) cells to chemotherapy by inhibiting mitophagy [133]. In contrast, the oncogene *MYC* uORF-encoded peptide MPEP promotes GBM cell growth by activating tropomyosin receptor kinase B (TRKB) and AKT-mTOR signaling; the overexpression of *MPEP* is related to the poor prognosis of GBM patients [134]. Notably, treatments with MPEP-neutralizing antibodies and TRKB inhibitors restrict GBM cell growth in the PDX model [134]. Moreover, uORF-encoded proteins can serve as potential neoantigens [135]. Considered together, these findings suggest uORFs are promising targets for therapeutics.

## 6 Conclusion

This review introduced detailed uORF mechanisms and factors contributing to uORF functions, while also summarizing uORF regulatory roles. Interestingly, uORF functions are conserved from microbes to higher eukaryotes. However, the substantial diversity in the underlying molecular mechanisms has been revealed by thorough examinations of uORFs in various microorganisms. Additionally, we briefly introduced the results of studies on uORFs in mammalian cells, which suggest that uORFs regulating the expression of particular genes may be potential therapeutic targets.

## Declaration of Competing Interest

All the authors declare no conflict of interest.
